# 3D-Printed Melatonin Tablets with Braille Motifs for the Visually Impaired

**DOI:** 10.3390/ph17081017

**Published:** 2024-08-01

**Authors:** Chrystalla Protopapa, Angeliki Siamidi, Aikaterini Sakellaropoulou, Siva Kolipaka, Laura Andrade Junqueira, Atabak Ghanizadeh Tabriz, Dennis Douroumis, Marilena Vlachou

**Affiliations:** 1 Section of Pharmaceutical Technology, Department of Pharmacy, National and Kapodistrian University of Athens, 15784 Athens, Greece; cprotopapa@pharm.uoa.gr (C.P.); asiamidi@pharm.uoa.gr (A.S.); 2 Section of Pharmaceutical Chemistry, Department of Pharmacy, National and Kapodistrian University of Athens, 15784 Athens, Greece; aiksakell@pharm.uoa.gr; 3 Centre for Research Innovation, University of Greenwich, Medway Campus, Chatham ME4 4TB, UK; s.kolipaka@gre.ac.uk (S.K.); ld3353f@gre.ac.uk (L.A.J.); 4 School of Life Sciences, University of Nottingham, Nottingham NG7 2RD, UK; atabak.ghanizadehtabriz@nottingham.ac.uk; 5 Delta Pharmaceutics Ltd., 1-3 Manor Road, Chatham ME4 6AE, UK

**Keywords:** melatonin, modified release, LCD 3D-printing, 3D printing, formulation development, Braille patterns, visual impairment, pharmaceutical technology, personalized medicine

## Abstract

An innovative approach for creating customized dosage forms and supporting patient populations with specific requirements who need additional support to improve drug adherence is 3D printing. This work introduces liquid crystal display (LCD) 3D printing as a means of developing melatonin (MLT) tablets. For patients who are blind or visually challenged, Braille patterns were displayed on the tablet surface in addition to the optimization of printing hydrogel inks. Owing to the great printing accuracy, blind patients could validate the Braille patterns that provided the required information. Upon further examination MLT was found to be present in the photopolymerized resins in an amorphous state. The choice of poly(ethylene glycol)-diacrylate (PEGDA) with varying molecular weights and the inclusion of surfactants or solubilizers interfered with the photopolymerization of the resin, hence controlling the rates of MLT dissolution towards the sought sustained release. Nuclear magnetic resonance (NMR) analysis showed that photopolymerization of the PEGDA resins in the printed dosage forms has taken place. A small batch scale-up investigation showed that LCDs could print a significant number of tablets quickly—about twenty-four minutes.

## 1. Introduction

Visual impairment (VI) refers to a condition in which a person’s visual ability or field of vision is reduced and cannot be corrected with spectacles or contact lenses. Approximately 285 million individuals globally are affected by visual impairment, with 39 million among them being blind. This condition can manifest from birth, childhood, early adulthood, or later stages of life. The demographic predominantly affected are those aged 50 and above, constituting 82% of the blind and visually impaired population [1,2]. People with visual impairment or blindness are usually disregarded in terms of obtaining proper medical care [3]. In comparison to the general population, these patients face an increased risk of patient safety incidents, as they are faced with a higher risk of medication errors [4]. This could be due to a variety of factors, including an inability to open medication containers, an inability to distinguish between different types of tablets/capsule dosage forms, forgetting to take medication on time, or taking the incorrect medication [5,6]. Medicine administration is complex and necessitates a variety of coordinated efforts on the part of both the patients and their caregiver(s) or family [7,8]. Many solutions to overcome these issues have been proposed and adapted from the visually impaired patients, such as medication management services, audio prescription labeling systems, special containers with Braille imprints, microchips embedded onto a prescription bottle that stores prescription labels and leaflet data using text-to-speech technology and mobile health applications that give equal opportunity for visually impaired patients to access health information [5,9,10]. Another way to deal with this issue is tablets with different shapes or imprints on them. To this extent, researchers have recently formulated and successfully produced three-dimensional printed oral dosage forms with Braille and Moon patterns for visually impaired patients [11].

Most sighted, visually impaired, and fully blind adolescents and adults experience natural 23.56 to 24.70 h cycles in their days [12]. The difference is that in sighted people, daily exposure to sunlight automatically resets cycle length to the 24-h day, while more than half of totally blind people have a 24.5-h circadian cycle [13]. This “free-running circadian rhythms” phenomenon causes them to stray from the actual time clock progressively. The exogenous administration of the chronobiotic hormone melatonin (MLT) mimicking its physiological secretion and typically taken in the late afternoon or early evening may prove to be the most effective approach to re-entrain the disarrayed circadian rhythms. The biosynthesis of MLT occurs at night, in the absence of light. Although the pineal gland is the primary MLT producer, research has revealed that MLT traces are also present in the retina, leukocytes, and the gastrointestinal system. Using tryptamine as a starting point, serotonin is formed during MLT’s biosynthesis process, the two final stages of which involve the *N*-acetylation and the *O*-methylation of serotonin. Amongst its many functions lies its capability to regulate the circadian rhythm [14]. The activation of MLT receptors divided into high (MT1, MT2) and low (MT3) affinity mediate this effect. Due to its advantages, MLT has been used to treat a variety of sleeping problems, including delayed sleep phase syndrome, jet lag, trouble falling and/or staying asleep, and sleep disorders related to the circadian rhythm in blind individuals [15].

A primary concern when formulating this hormone is the determination of the optimum dose for each individual user, since MLT reaction varies depending on individual circadian time, and duration of usage [13]. Also, various melatonin formulations, including fast release, modified release, or bimodal release, may have different effects. As MLT has a short half-life and poor bioavailability, sustained-release formulations mimic the physiological MLT release rate closer to the fast ones [16]. Dose modifications and changes in formulations must be made, creating stress, pain, and a lack of therapy benefit for the patient. A rising amount of published research shows that it is vital to shift away from the conventional ‘one-size-fits-all’ strategy and personalize the therapy to the individual’s needs, delivering a more patient-centric approach [17]. For this reason, 3D printing technology can be used for drug product development of customized medicines with high accuracy and reproducibility [18]. MLT 3D-printed tablets can be fabricated according to the patient’s needs, i.e., personalized shape, size, formulation type, dose, multi-drug combinations, and tailored release profiles [19]. Several articles have emerged using MLT and 3D printing technology. Recently, researchers have produced 3D-printed MLT esophageal stents with stereolithography (SLA) technology that showed long retention and sustained MLT release in the esophagus [20]. In another work, 3D-printed LEGO^®^-like tablets were printed with fused deposition modeling using MLT and caffeine with customized release kinetics that can potentially be used for the treatment of sleep disorders [21]. Scientists have also developed a melatonin-loaded 3D-printed magnesium–polycaprolactone scaffold and investigated its effects and molecular mechanism on inhibiting osteosarcoma growth and metastasis [22]. To the best of our knowledge, it is the first time that melatonin was used for the preparation of 3D-printed tablets with the aid of liquid crystal display (LCD) printers as a promising application in the treatment of sleep problems.

Light-cured 3D printing offers several benefits over other printing techniques, such as high accuracy, quick forming speed, and low cost [23]. SLA printers use light-curing technology to create a 3D-printed tablet layer by layer, employing an ultraviolet laser as the light source. The laser point is precisely controlled by a spinning mirror to scan the cross-sectional outlines, curing one layer after another. The primary benefit of this technology is increased precision and print quality, since the laser’s precise movement and small size allow for greater detail and resolution. This increased quality comes at the expense of print speed; thus, sketching each layer might be time-consuming [24]. On the other hand, digital light processing (DLP), which is the next generation of SLA printers, is a less time-consuming 3D printing method as it employs a projected light source to cure the entire layer at once [23]. LCD 3D printers use the same basic principle: they illuminate a cross-section of the 3D print by masking the UV light source with an LCD screen. This particular printer technology uses a light source to solidify a photopolymer resin. Specifically, the light source (comprised of ultraviolet light-emitting diodes—UV LEDs) shines through an LCD screen and cures the photosensitive resin, which is stored in a tank creating the 3D-printed tablet layer-by-layer on a platform. This construction platform slowly rises from the tank, which is filled with resin, as the 3D-printed tablet is formed [25].

Over the last fifteen years, our research team has been developing novel oral MLT dosage forms for the treatment of sleep onset and maintenance disorders [26,27,28]. The scope of this study was to investigate the feasibility of formulating personalized MLT 3D-printed tablets, using a Halot Sky 2022 LCD 3D printer, encompassing Braille-imprinted patterns, and reading MLT for easy identification by visually impaired individuals. Moreover, a sustained MLT release profile was sought, as the circadian release of endogenous MLT in these patients is disrupted by either penumbra (visually impaired) or darkness (blind people).

## 2. Results

### 2.1. Printing Process

**Figure 1:**
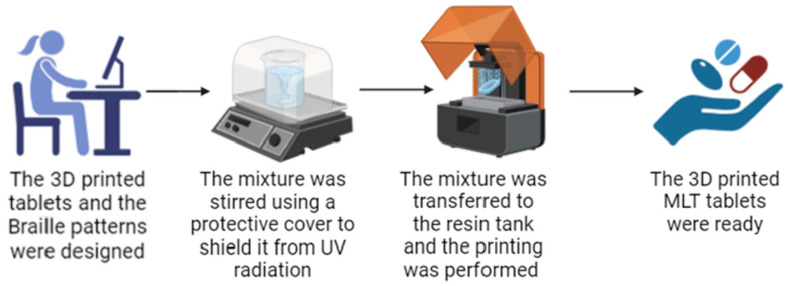
The printing process of the MLT 3D-printed tablets with the Braille patterns. The figure was generated using BioRender.com (http://www.biorender.com/, accessed on 9 April 2024).

**Figure 2:**
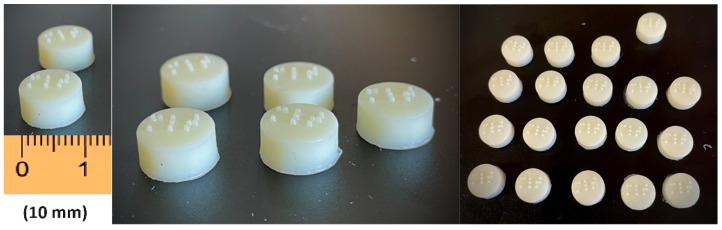
The MLT 3D-printed tablets with Braille patterns.

As shown in Figure 1, for this work, LCD 3D printing technology was successfully used to imprint Braille patterns onto the surface of cylindrical 3D-printed tablets (composition shown in Table 1) that were evaluated with tactile recognition by a blind member of the Panhellenic Society for the Visually Impaired. This study aimed to develop MLT-personalized oral solid dose forms, which were specifically customized for blind or visually impaired patients. The photos of the MLT 3D-printed tablets (shown in Figure 2) notably demonstrate the capacity of the LCD 3D printer to produce tablets with intricate and complicated patterns.

The settings in Table 2 were fully optimized by adjusting various printing parameters such as layer thickness, initial exposure, and exposure time for each layer. The optimized printing improved tablet features such as hardness and friability including the printing accuracy. Overall, PEGDA is widely utilized in numerous biomedical applications because it is cytocompatible, non-toxic, and user-friendly [29,30,31]. Many researchers have used PEGDA in the development of oral delivery formulations [25,32,33,34,35,36,37,38]. However, to date, there has been no toxicological research confirming the safety and non-toxicity of PEGDA following oral administration. In this study, two different molecular weights of PEGDA, PEGDA400 and PEGDA700, were utilized. The increase in molecular weight of PEGDA affects the drug release rate and swelling ratio, which will be discussed later in the article.

The other two excipients, PEG200 and Tween 80, were used in varying ratios to regulate the release of MLT from the printed tablets and differently affect the swelling ratio. When combined with PEGDA, these excipients interfere with the polymerization process. Specifically, in the presence of the excipient molecules, polymerization leads to the formation of holes. These holes enhance the dissolution and release of the drug into the dissolution medium.

A major feature of the LCD printing technology is the rapid printing times in comparison to SLA. By taking advantage of this property, we fabricated a small-scale batch that could potentially meet the needs of a hospital ward or a pharmacy. As shown in Figure 2, it was feasible to print tablets featuring Braille patterns with high accuracy and reproducibility. Most importantly, LCD achieved the printing of 63 tablets within only 24 min; hence, the process can be repeatedly performed to produce a sufficient amount of tablets by using a single printer.

Furthermore, the easiness of producing printed tablets with Braille motifs and different dosage forms (not studied here) renders the LCD technology ideal for manufacturing medicines at points of care such as hospitals and pharmacies.

### 2.2. Characterization of 3D-Printed Tablets

The 3D-printed tablets were evaluated regarding their dimensions and weights. As shown in Table 3, the dimensions of the tablets appeared to be consistent with the initially set inputs for the printing process (target dimensions: x = 10 mm, y = 10 mm, z = 5.2 mm). The mechanical properties of 3D-printed tablets were examined; the formulations containing PEGDA400 (F1 and F2) showed higher breaking force in comparison to those comprising PEGDA700 (F3 and F4). From the recorded values, it is evident that the molecular weight of the PEGDA plays a key role in the final tablet hardness. The low molecular weight PAGDA produced stronger and stiffer tablets regardless of the presence of Tween 80 or PEG200.

### 2.3. Physicochemical Characterization

**Figure 3:**
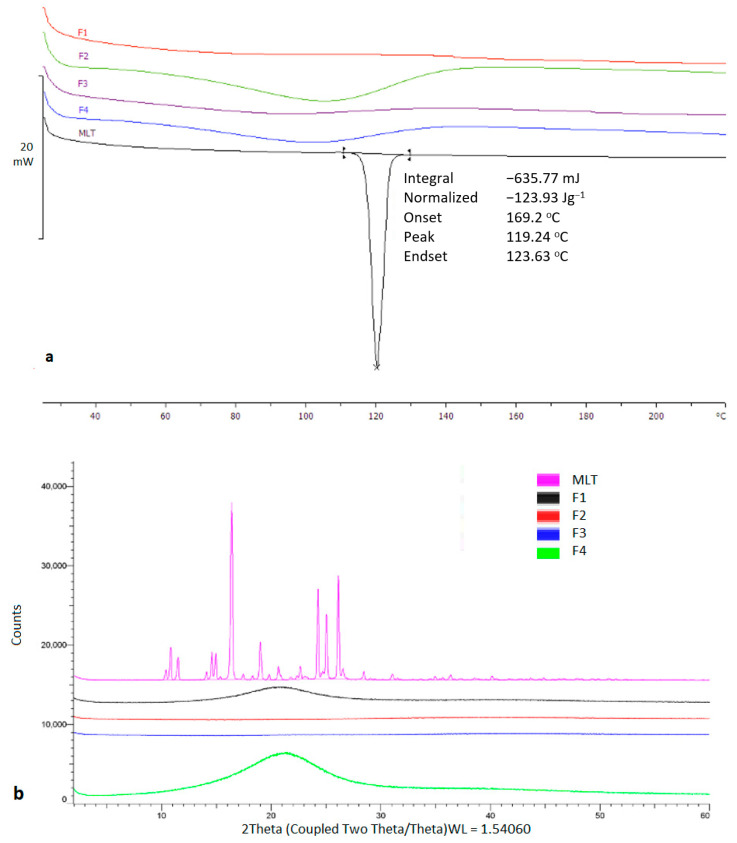
(**a**) DSC thermogram of bulk TMLT and printed tablets, (**b**) X-ray Diffraction of bulk TMLT and printed tablets.

Thermal analysis was carried out to investigate the physical state of MLT prior and after the printing process. As shown in Figure 3, the bulk MLT presented a sharp melting endotherm at 116.92 °C. On the contrary, MLT appeared to be in an amorphous state within the printed tablets.

The observed results were also confirmed by X-ray analysis which showed distinct diffraction peaks for bulk MLT at 10.4°, 10.8°, 15.3°, 16.4°, 17.4°, 24.2°, 25.0°, and 26.1° 2theta values, which were eliminated in the printed structures. MLT is highly miscible in PEG200 and Tween 80, while it is freely soluble in water, hence the presence of amorphous content was anticipated.

### 2.4. ^1^H-NMR Spectral Analysis

**Figure 4:**
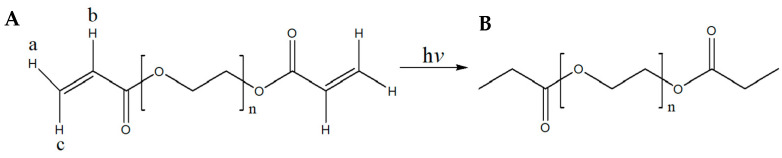
(**A**) Structure of PEGDA400 and PEGDA700; (**B**) Structure of UV irradiated PEGDA400 and PEGDA700.

From Figure 4, it becomes apparent that the omega (*ω*) *α*,*β*-unsaturated carbonyl moiety of PEGDA400 (Figure 4A) used as the photopolymerizable monomer is transformed upon UV irradiation into its respective saturated carbonyl group (Figure 4B). In detail, the olefinic protons a, b, and c, resonate at 5.95 (1H, dd) ppm, 6.19 (1H, dd) ppm, and 6.33 (1H, dd) ppm, respectively (Figure S1A see Supplementary Materials). The coupling constants of proton a are: *J*_gem_ = 1.64 and *J*_trans_ = 10.25 Hz, of b: *J*_cis_ = 10.24 Hz and *J*_trans_ = 17.27 Hz and of c: *J*_gem_ = 1.64 Hz and *J*_trans_ = 17.27 Hz. These protons are not present in the spectrum of Figure S1B.

Similarly, from Figure S2 (see Supplementary Materials), it becomes apparent that the omega (*ω*) *α*,*β*-unsaturated carbonyl moiety of PEGDA700 (Figure 4A), used as the photopolymerizable monomer, is transformed, upon UV irradiation, into the respective saturated carbonyl group (Figure 4B). In detail, the olefinic protons a, b, and c, resonate at 5.95 (1H, dd) ppm, 6.19 (1H, dd) ppm, and 6.34 (1H, dd) ppm, respectively (Figure S2A see Supplementary Materials). The coupling constants of proton a are: *J*_gem_ = 1.62 and *J*_trans_ = 10.25 Hz, of b: *J*_cis_ = 10.25 Hz and *J*_trans_ = 17.27 Hz and of c: *J*_gem_ = 1.62 Hz and *J*_trans_ = 17.27 Hz. These peaks were not present after PEGDA700 was photopolymerized (Figure S2B see Supplementary Materials). Moreover, Figures S1 and S2 (see Supplementary Materials) clearly show that under the described irradiation conditions, compounds PEGDA400 and PEGDA700, have been converted to their saturated congeners, respectively. Last, in Figure S3 (see Supplementary Materials), it is clearly shown that the chemical structure of MLT remained intact during the fabrication of the F1 and F4 printlets.

### 2.5. SEM Image Analysis

**Figure 5:**
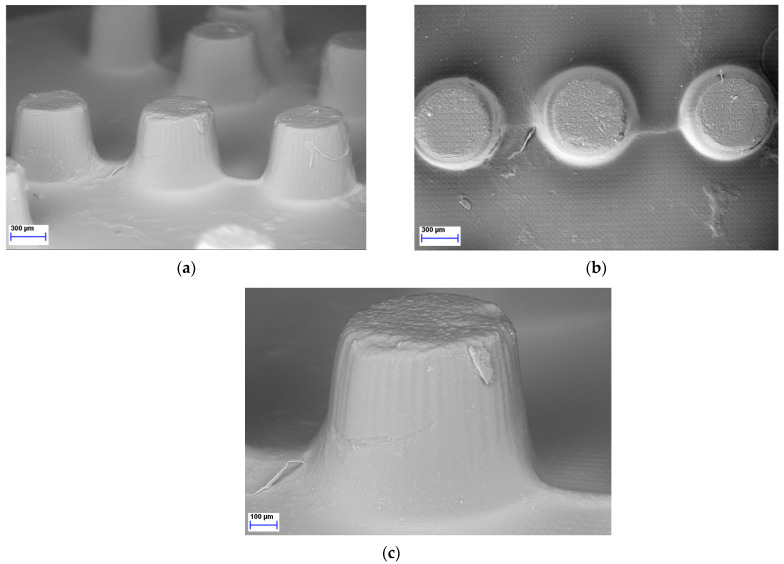
SEM figures of MLT 3D-printed tablets showing the Braille imprints: (**a**) Side view, magnification ×37; (**b**) Top view, magnification ×38; (**c**) Side view, magnification ×85.

As the LCD 3D printer was effectively used to print Braille patterns on the upper surface of the tablets containing MLT it was imperative to further analyze the morphology and resolution of printed structures. Hence, SEM was performed to analyze the microstructure of the photopolymerized ink formulations, resulting in Braille patterns. As seen in Figure 5, each dot/character of the Braille patterns on the surface of the 3D-printed tablets was detailed, allowing for tactile identification.

### 2.6. Dissolution Studies

**Figure 6:**
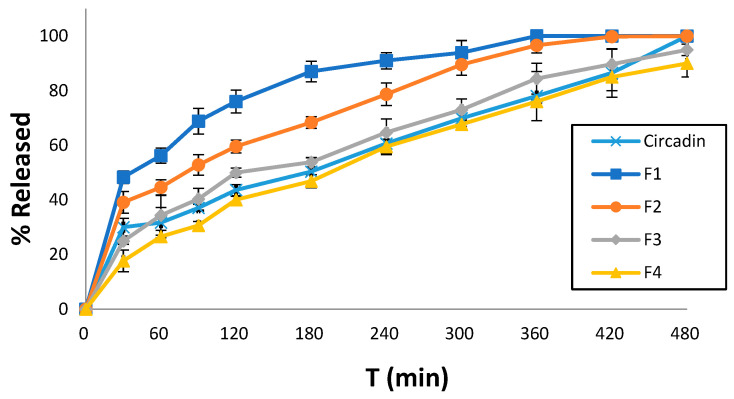
Comparative MLT release rates of four printed tablet formulations and the commercially available Circadin^®^ vs. time (min) at pH 4.5. Results represent the mean value (*n* = 3, SD < 2).

Dissolution studies were conducted for all printed tablet formulations to identify the effect of the excipients used in each resin ink. Notably, at the end of the experimental procedure (480 min), the 3D-printed tablets were found preserved at the bottom of the dissolution vessel. All formulations showed sustained release profiles, which decreased in an ascending order of F1 > F2 > F3 > F4. Specifically, F1 showed 76% MLT release within 120 min followed by complete release after 7 h. Similarly, F2 showed 99% MLT release after 7 h while for F3 and F4, the rates were significantly lower to 85%, respectively. As shown in Figure 6, the release profiles of F3 and F4 are comparable to the commercially available Circadin^®^. Hence, it is evident that the careful selection of resin formulations and optimization of the printing process can match the dissolution profiles of marketed products.

The MLT strength in the 3D-printed tablets was found to be 2 mg equal to the theoretical loading in the printable inks.

### 2.7. Mathematical and Statistical Analysis

Table 4 summarizes the release kinetics of MLT from the 3D-printed formulations. In most cases, the release mechanism follows the zero-order model, providing a constant release rate over time. At the same time, the release kinetics of F3 and F4 can also be explained with the Higuchi and Korsmeyer–Peppas equations as well [39].

The *n* value of the Korsmeyer–Peppas equation indicates that MLT’s release from F1 and F2 follows the Fickian diffusion model (n ≤ 0.45), whereas from F3 and F5 an anomalous diffusion release is followed (0.45 ≤ n ≤ 0.89).

### 2.8. Determination of Swelling Ratio (Q)

**Figure 7:**
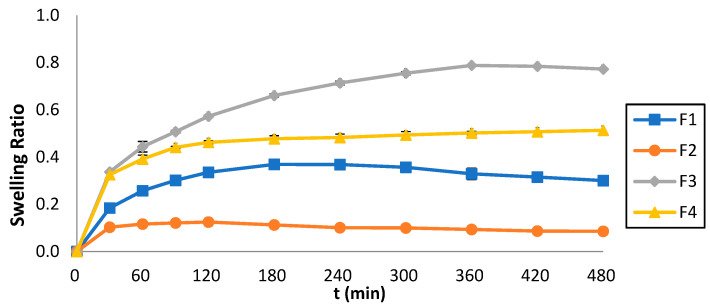
Swelling ratios of the 3D-printed tablets vs. time.

Figure 7 illustrates the swelling ratio (Q) of the 3D-printed tablets which was monitored over 8 h, and the Q vs. time was plotted. The maximum swelling ratio for F1 was 0.37 shown at t = 180 min, for F2 it was 0.13 shown at t = 120 min, for F3 it was 0.79 shown at t = 360 min, whereas for F4 it was 0.51 shown at t = 480 min. The swelling behavior was strongly affected by the ink composition. By comparing Figure 6 and Figure 7, it can also be observed that lower swelling rates were accompanied by faster dissolution rates.

## 3. Discussion

Although SLA printers have been widely used in pharmaceutics, LCD printing is quite young, having just debuted in recent years and just two groups of researchers have used this technology for oral solid dosage forms. To this extent, scientists have successfully printed extended-release 3D-printed tablets [25] and placebos of vortioxetine hydrobromide tablets [40]. The current work demonstrates the feasibility of producing a two months’ supply (given the dose is one tablet at nighttime) of MLT 3D-printed tablets customized for the VI patients with Braille identification patterns in less than 30 min.

The successful printing process can also be verified by the uniformity of the 3D-printed tablets’ physical characteristics and the SEM imaging analysis that concluded effective Braille pattern formation. Regarding the drug content, all formulations of the 3D-printed tablets exhibited MLT concentration close to 100%, indicating homogeneity for the tablets and a reproducible printing method.

As mentioned in Section 2.4, in the ^1^H-NMR spectra of both PEGDA400 (Figure S1A Supplementary Materials) and PEGDA700 (Figure S2A Supplementary Materials), the six olefinic protons appear as three doublets of doublets at 5.95–6.33 ppm. From the ^1^H-NMR spectra of the UV-irradiated PEGDA400 (Figure S1B Supplementary Materials) and PEGDA700 (Figure S2B Supplementary Materials), it becomes apparent that these olefinic protons are not present. Analogous observations have been reported by Imani et al. [41]. Moreover, the spectra of the 3D-printed tablets F1 and F4 (Figure S3) show that the chemical structure of melatonin remained intact during their fabrication.

For the release profile of MLT from the 3D-printed F3 and F4 formulations, which is comparable to that of the commercially available drug Circadin^®^ (Figure 6), it could be argued that the presence of PEGDA700 in their formulation leads to a slower MLT release. Conversely, the release of MLT from the PEGDA400 containing F1 and F2 formulations is notably higher, especially in the case of F1, due to the presence of the solubilizer surfactant Tweeng 80, compared to PEG200 present in the F2 formulation. Overall, the release behavior of drug molecules is complex, and it depends on several factors. Usually, the higher molecular mass induces increased polymer–water interactions, increased swelling, and decreased crosslinked density, which in turn facilitates faster release rates. However, it is not unusual that PEGDA with lower molecular mass results in faster dissolution rates (e.g., proteins). This has been attributed to potential interactions with other excipients such as PEG200 or Tween 80, which can form non-covalent bonds, or to the higher viscosity of PEGDA700.

Although there are minor toxicity concerns related to PEGDA400 monomers, due to the acrylic functions located at both ends of the chain, its full polymerization using photoinitiators, which is well documented [42], obliterates these issues. This, accompanied by the fact that the ^1^H-NMR spectrum of the photoirradiated PEGDA400 monomer did not show any resonating olefinic protons, suggests that the polymerized PEGDA400 employed in the present work is devoid of the presence of any hazardous oligomers. Yet, as it is very important to be cautious with the use of PEGDA400, it is advised that thorough checks be conducted, irrespectively of the method employed for its polymerization (e.g., electron beam irradiation or the use of thermal initiators).

Regarding the Q, as previously stated, the results illustrate that the formulations containing PEGDA700 exhibited greater Q values (F3 = 0.79 and F4 = 0.51) compared to those containing PEGDA400 (F1 = 0.37 and F2 = 0.31). This pattern can be attributed to the molecular weight of PEGDA; the higher the molecular weight, the stronger the swelling effects. Additionally, the formulations containing Tween 80 (F1 and F3) exhibit higher Q values than those containing PEG200 (F2 and F4). This behavior can be related to the interaction of Tween 80 with the aqueous media, generating a spindle-shaped ellipsoid structure, which promotes swelling [35], as opposed to PEG200.

## 4. Materials and Methods

### 4.1. Materials

The active ingredient melatonin (MLT), the excipients polyethylene glycol (PEG200, average MW 200), polysorbate 80 (Tween 80), polyethylene glycol diacrylate 400 (PEGDA400, average MW 400), and the photoinitiator phenyl-bis(2,4,6-trimethylbenzoyl)phosphine oxide (TPO) were purchased from the Tokyo Chemical Ltd. (Tokyo, Japan). The excipient polyethylene glycol diacrylate 700 (PEGDA700, average MW 700) was purchased from Sigma–Aldrich Ltd. (Steinheim, Germany). The commercially available medicine Circadin^®^ was purchased from a local pharmacy store.

### 4.2. Three-Dimensional Printing

Each one of the four formulations was prepared by dissolving MLT in water, under continuous stirring, at room temperature. The target dose of MLT for each tablet was 2 mg. The combination of excipients as shown in Table 1; PEG200, Tween 80, and either PEGDA400 or PEGDA700 (used as the photopolymerizable monomer) were then incorporated into the solution and stirred in an IKA^®^ RCT standard magnetic stirrer (LLG Labware, Meckenheim, Germany) for 10 more min to ensure uniformity. The photoinitiator TPO was subsequently added under stirring, for approximately another 4 h until the TPO was fully dissolved. To ensure that no premature solidification occurred, a protective cover was used to shield the mixing procedure from the UV radiation. The prepared mixture was then used for the printing process. All of the 3D-printed tablets were prepared using a Halot Sky 2022 LCD 3D printer (Creality, Shenzhen, China). The printer laser (405 nm) was capable of producing items with a resolution precision of 0.034 smm, layer thicknesses of 0.01–0.2 mm, and printing speeds of 1–4 s/layer. The Duxbury Braille translator Win 12.7 SR2, 2023, was used to convert the letters MLT (used for melatonin) into Braille patterns. The 3D-printed tablets (*x* = 10 mm, *y* = 10 mm, *z* = 5.2 mm) with the Braille pattern (*x* = 0.7 mm, *y* = 0.7 mm, and *z* = 0.5 mm) were created in AutoCAD^®^ 2023 (Autodesk Inc, San Rafael, CA, USA) and exported as a stereolithographic file (.stl). The formulation was added to the resin tray, while the printer’s operational settings were adjusted in accordance with Table 2.

The tablets were printed straight on the building platform at an ambient temperature with no supports. The 3D-printed tablets were washed with deionized water, blotted with filter paper to remove any uncured liquid formulation, and then UV-cured for an additional 2 min to guarantee full polymerization.

### 4.3. Physical Properties and Breaking Force of the 3D-printed Tablets

The 3D-printed tablets were weighed using a precise 4-digit scale (ATX224, Shimadzu, Kyoto, Japan) and their dimensions (both width and height) were measured using a Vernier caliper. To determine the breaking force of the printed tablets, a hardness tester (TBH 28, Erweka GmbH, Heusenstamm, Germany) was used. The process involved adjusting stress perpendicular to the tablet’s axis on its opposing sides till it fractured.

### 4.4. X-ray Diffraction (XRPD)

X-ray diffraction analysis of MLT and 3D-printed tablets (x = 10 mm, y = 10 mm, z = 5.2 mm) was conducted using a D8 Advance diffractometer (Bruker, Billerica, MA, USA) equipped with Cu Kα radiation (40 kV, 40 mA) and a LynxEye silicon strip detector. Data were collected with a step size of 0.02° and a counting time of 0.3 s per step, covering an angular range from 3 to 40° 2θ at a speed of 2° per minute. The applied power was 15 mA, and the voltage was 40 kV.

### 4.5. Differential Scanning Calorimetry (DSC)

The thermal behavior of MLT was analyzed through a DSC-3 (Mettler Toledo, Greifensee, Switzerland). Each sample (4–5 mg) was precisely weighed in aluminum pans, which were then carefully sealed with lids. Tests were conducted on pure MLT and four different formulations. The DSC experiments ranged from 25 °C to 250 °C, with a heating speed of 10 °C/min and a continuous nitrogen gas flow at 20 mL/min. As a reference, an empty aluminum pan was used.

### 4.6. ^1^H-NMR Spectral Analysis

The ^1^H-NMR spectra of the PEGDA400 and PEGDA700, the UV-irradiated PEGDA400 and PEGDA700, and those of the F1 and F4 printlets were taken in deuterated DMSO-d6 and recorded on a Bruker DRX 400 (400 MHz) spectrometer (Karlsruhe, Germany); the chemical shifts are presented in ppm. The ^1^H-NMR PEGDA400 and PEGDA700 samples were prepared by diluting 0.05 mL of the corresponding PEGDA compound in DMSO-d6 (0.6 mL). The corresponding irradiated ^1^H-NMR PEGDA samples were prepared by dissolving 2 mg of the polymerized material in DMSO-d6 (0.6 mL). For the preparation of the NMR samples of printlets F1 and F4, the respective disks were treated with DMSO-d6 (0.7 mL) under sonication conditions for 30 min. The cloudy solution formed was filtered through a 0.45 μm pore size filter (Millipore Ltd., Ireland), and the filtrate was poured onto an NMR tube to record the respective spectra.

### 4.7. SEM Image Analysis

Scanning electron microscopy (Hitachi SU8030, Tokyo, Japan) was used to evaluate the Braille-imprinted patterns on the 3D-printed tablets’ morphology. The SEM pictures were taken with an electron beam accelerating voltage of 2 kV and magnifications of 37, 38, and 85×.

### 4.8. Determination of MLT Tablet Strength

The 3D-printed tablets were crushed with a mortar and pestle before being dissolved in deionized water (1 L) and stirred with a magnetic stirrer for 24 h. Samples were filtered via 0.45 μm filters (Millipore Ltd., Dublin, Ireland) and the MLT concentration was measured with a UV-VIS spectrophotometer (uniSPEC 2 Spectrophotometer LLG Labware, Meckenheim, Germany) at λ_max_ = 278 nm. The measurements were repeated three times to ensure accuracy.

### 4.9. Dissolution Studies

The MLT release from each 3D-printed formulation and the commercially available Circadin*^®^* was assessed through a USP II dissolution paddle apparatus (PharmaTest-D17, Hainburg, Germany) in a pH 4.5 aqueous medium at 50 rpm and 37 ± 0.5 °C. Samples of 5 mL were taken at fixed time intervals, filtered using 0.45 μm pore size filters (Millipore Ltd., Dublin, Ireland), and analyzed with a UV-VIS spectrophotometer (uniSPEC 2 Spectrophotometer LLG Labware, Meckenheim, Germany) at λ_max_ = 278 nm. The dissolution tests were conducted three times, and graphs depicting the percentage release (mean ± SD) over time were created.

### 4.10. Mathematical and Statistical Analysis

The data gathered from the dissolution tests were fitted to different release kinetics including first- and zero-order, Higuchi and the Korsmeyer, using the GraphPad Prism 8 (version 5.01) [39,43].

### 4.11. Determination of Swelling Ratio (Q)

Three 3D-printed tablets (Md dried state) from each formulation were weighed, and placed into a pH 4.5 aqueous medium for 8 h at 37 ± 0.5 °C. At predetermined time points, the excess water was carefully wiped off and the swelled 3D-printed tablets were weighed again (Ms) [44]. The Q was calculated using the following equation:

(1) $$Q=\frac{Ms-Md}{Md}$$

## 5. Conclusions

To create customized MLT tablets with Braille patterns for patients who are blind or visually impaired, LCD printing technology was implemented. The key factors to attaining sustained drug release rates for more than 8 h were concluded to be the composition of printing inks, comprising PEGDA of various molecular weights, and the presence of solvents or surfactants. When combined with PEGDA, the utilized excipients interfere with the polymerization process, leading to the formation of holes. These holes enhance the dissolution and release of the drug into the dissolution medium, achieving optimal dissolution rates of MLT. Although NMR tests showed that the produced 3D-printed tablets achieved complete polymerization, there is currently no toxicological research certifying the safety and non-toxicity of PEGDA excipients when administered orally. As a result, further investigation is needed to ensure that there are no toxic effects on the body both in the short term and in the long term following its administration. LCD printing technology can be a valuable tool for the development of personalized dosage forms at the point of care due to fast printing times with high accuracy, thus it should be further exploited.

## Figures and Tables

**Table 1:** Composition of the developed 3D-printed tablets.

Formulation	F1 (%*w*/*w*)	F2 (%*w*/*w*)	F3 (%*w*/*w*)	F4 (%*w*/*w*)
MLT	0.5	0.5	0.5	0.5
PEGDA400	28.21	28.21		
PEGDA700			28.21	28.21
Tween80	56.59		56.59	
PEG200		56.59		56.59
H_2_O	13.7	13.7	13.7	13.7
TPO	1	1	1	1

**Table 2:** Printing parameters set on the LCD 3D printer.

Parameters	Values
Layer thickness	0.050 mm
Initial exposure	40 s
Exposure time	3 s
Rising high	8 mm
Motor speed	5 mm/s
Turn off delay	4 s
Bottom exposure layer	2

**Table 3:** Physical characteristics of the 3D-printed tablets (*n* = 10).

Formulations	Width (mm)	Height (mm)	Weight (mg)	Hardness (N)
F1	10 ± 0.32	5.2 ± 0.23	398.34 ± 2.37	5.07 ± 0.48
F2	10 ± 0.44	5.2 ± 0.30	408.33 ± 1.16	4.88 ± 0.81
F3	10 ± 0.51	5.2 ± 0.13	378 ± 3.56	2.37 ± 0.42
F4	10 ± 0.27	5.2 ± 0.15	415.67 ± 3.23	1.93 ± 0.30

**Table 4:** Results of fitting various models to drug release profiles from the 3D-printed tablets.

Formulation	Zero Order			First Order		Higuchi		Korsmeyer–Peppas		
	R^2^	Y_0_	K_0_	R^2^	Y_1_	R_2_	K_H_	R^2^	K_KP_	*n*
F1	0.90	52.05	0.15	0.85	56.38	0.62	6.01	0.90	14.35	0.35
F2	0.96	40.00	0.14	0.90	46.70	0.93	5.03	0.95	10.55	0.36
F3	0.98	26.27	0.15	0.93	34.33	0.99	4.32	0.95	4.47	0.49
F4	0.99	17.43	0.16	0.93	27.31	0.97	3.91	0.99	1.99	0.62

## Data Availability

Data is contained within the article or Supplementary Materials.

## Supplementary Materials

The following supporting information can be downloaded at: https://www.mdpi.com/article/10.3390/ph17081017/s1, Figure S1: ^1^H-NMR (400 MHz, DMSO-*d*_6_) of A: PEGDA400 and B: UV irradiated PEGDA400; Figure S2: ^1^H-NMR (400 MHz, DMSO-*d*_6_) of A: PEGDA700 and B: UV irradiated PEGDA700; Figure S3: ^1^H-NMR spectra (400 MHz, DMSO-*d*_6_) of MLT 3D printed tablets A: F1 and B: F4.
