# Supplementary material for: 3D-Printed Melatonin Tablets with Braille Motifs for the Visually Impaired
Source: Pharmaceuticals (Basel). 2024 Aug 1;17(8):1017. doi: 10.3390/ph17081017 (PMC11357011; doi:10.3390/ph17081017)
Supplement: Supplementary file 1 [file pharmaceuticals-17-01017-s001.zip › pharmaceuticals-3074785-supplementary.pdf]

Supplementary material

3D-Printed Melatonin Tablets with Braille Motifs for the Visually Impaired

Chrystalla Protopapa [1], Angeliki Siamidi [1], Aikaterini Sakellaropoulou [2], Siva Kolipaka [3], Laura Andrade Junqueira [3], Atabak Ghanizadeh Tabriz [4], Dennis Douroumis [3,5,*] and Marilena Vlachou [1,*]

[1] Section of Pharmaceutical Technology, Department of Pharmacy, National and Kapodistrian University of Athens, 15784 Athens, Greece; cprotopapa@pharm.uoa.gr (C.P.); asiamidi@pharm.uoa.gr (A.S.)
[2] Section of Pharmaceutical Chemistry, Department of Pharmacy, National and Kapodistrian University of Athens, 15784 Athens, Greece; aiksakell@pharm.uoa.gr
[3] Centre for Research Innovation, University of Greenwich, Medway Campus, Chatham ME4 4TB, UK; s.kolipaka@gre.ac.uk (S.K.); ld3353f@gre.ac.uk (L.A.J.)
[4] School of Life Sciences, University of Nottingham, Nottingham NG7 2RD, UK; atabak.ghanizadehtabriz@nottingham.ac.uk
[5] Delta Pharmaceutics Ltd., 1-3 Manor Road, Chatham ME4 6AE, UK
* Correspondence: d.douroumis@gre.ac.uk (D.D.); vlachou@pharm.uoa.gr (M.V.)

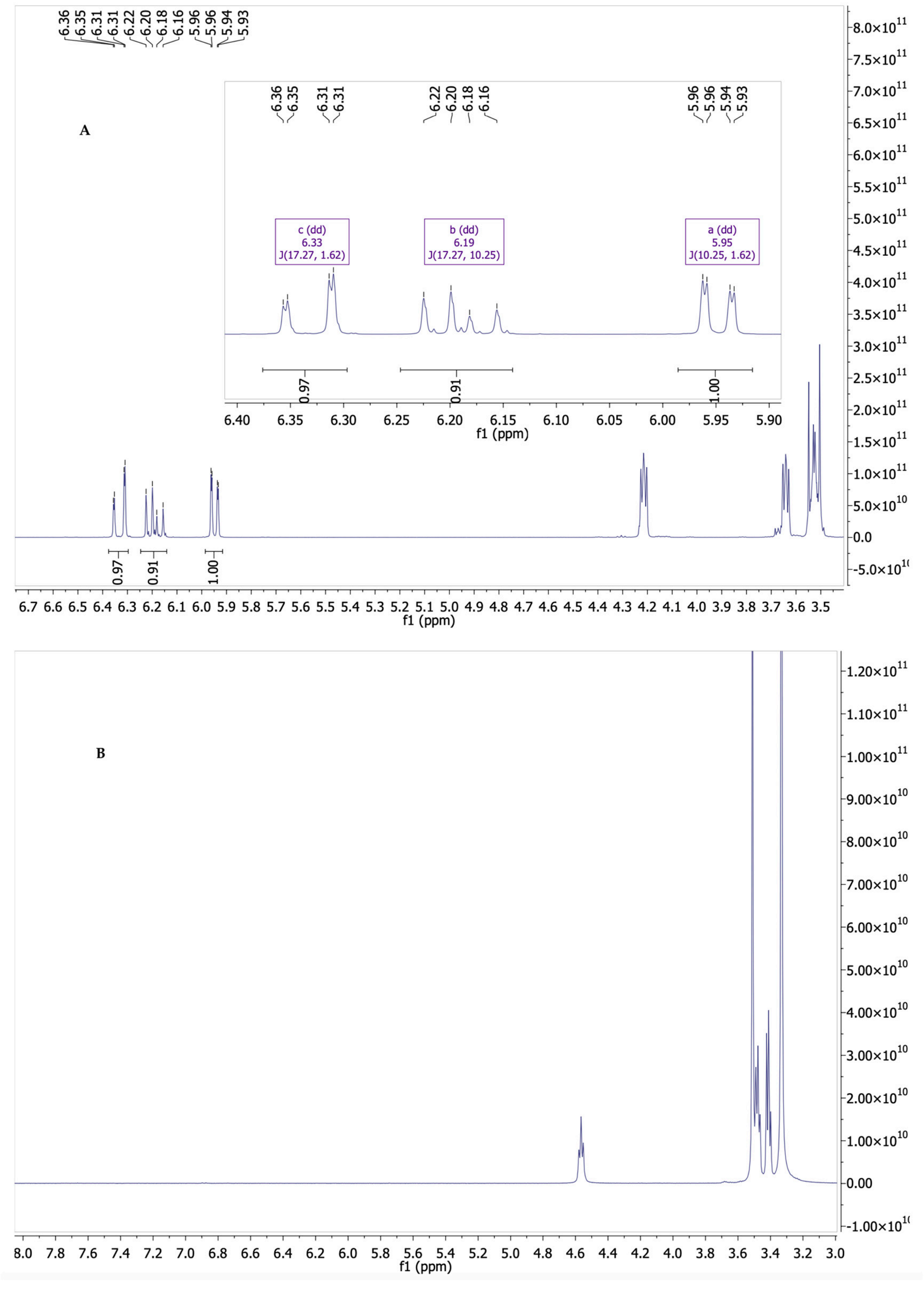

Figure S1. ^{1}H-NMR (400 MHz, DMSO-d_6) of A: PEGDA400 and B: UV irradiated PEGDA400

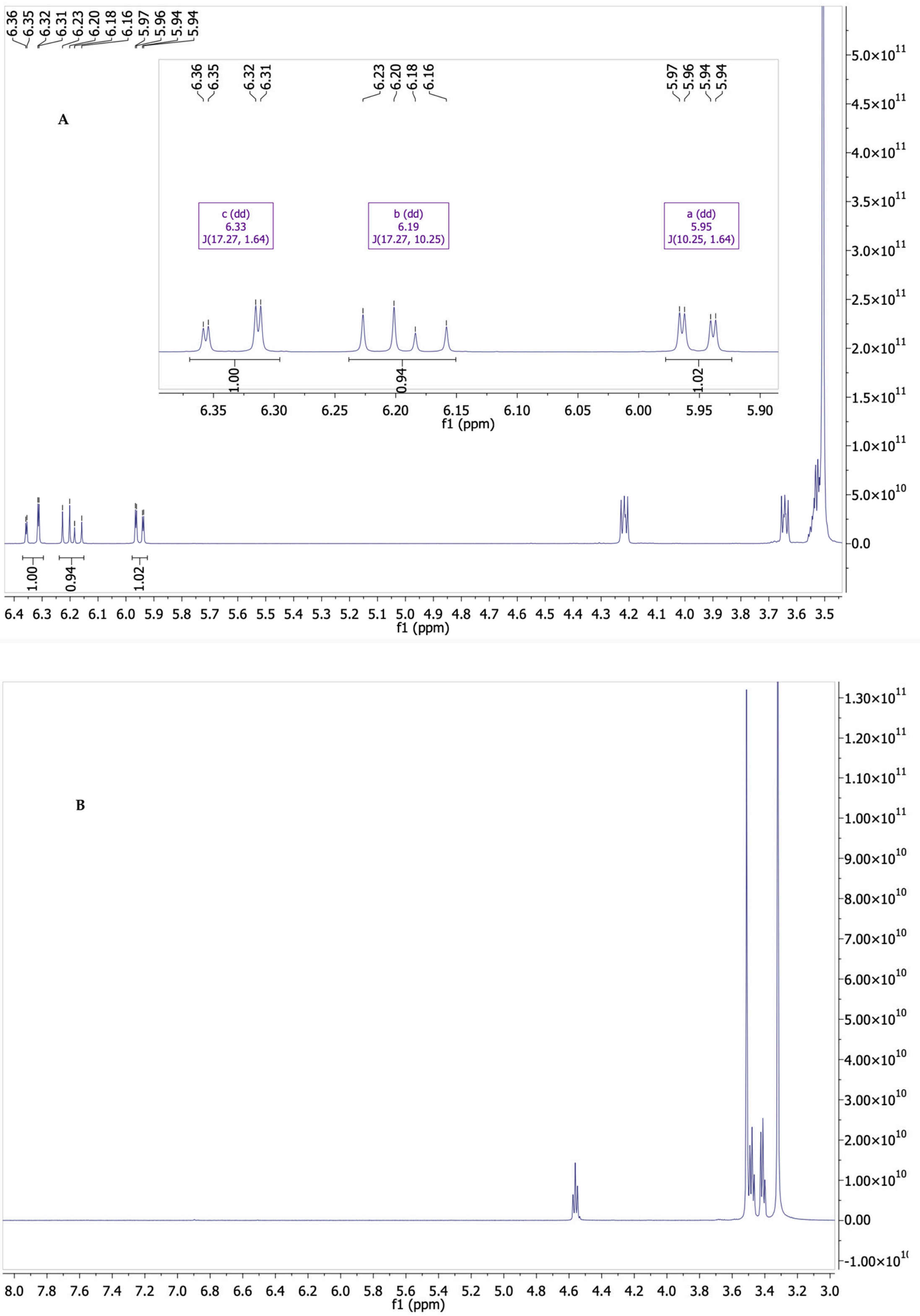

Figure S2. ^{1}H-NMR (400 MHz, DMSO-d_6) of A: PEGDA700 and B: UV irradiated PEGDA700

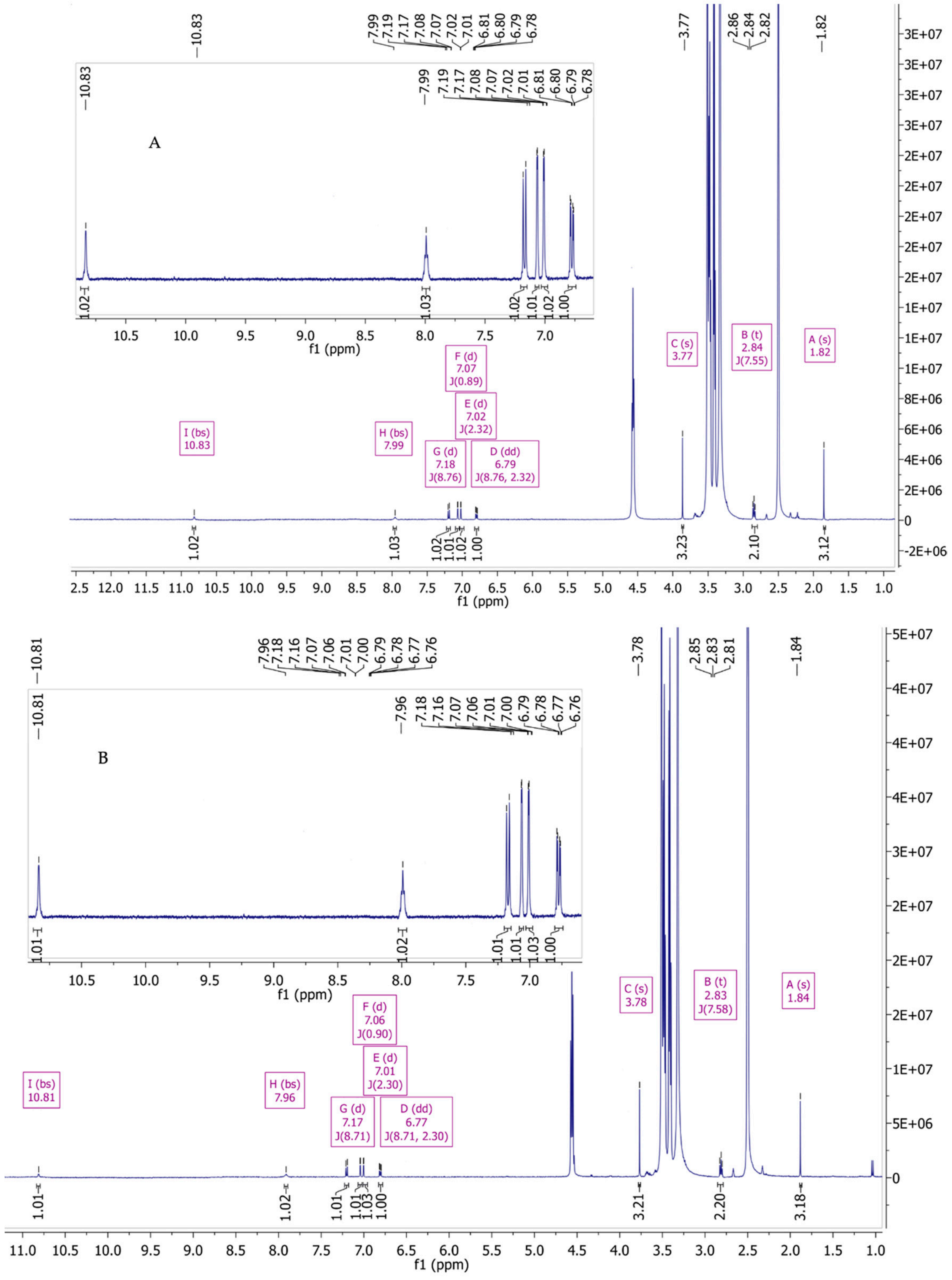

Figure S3: ^{1}H-NMR spectra (400 MHz, DMSO-d_6) of MLT 3D printed tablets A: F1 and B: F4
